# Environmental Pollution with Bisphenol A and Phthalates—A Serious Risk to Human and Animal Health

**DOI:** 10.3390/ijerph192113983

**Published:** 2022-10-27

**Authors:** Slawomir Gonkowski, Krystyna Makowska

**Affiliations:** Department of Clinical Physiology, Faculty of Veterinary Medicine, University of Warmia and Mazury, Oczapowskiego Str. 13, 10-718 Olsztyn, Poland

Pollution of the natural environment is constantly increasing due to industrial development and progressive urbanization. Among the many substances present in the natural environment due to human activity, bisphenol A (BPA) and phthalates deserve special attention due to their widespread use in industry and their multidirectional harmful effects on living organisms. BPA and phthalates are plasticizers—substances used in plastic production to increase the longevity, durability and flexibility of products [1,2]. Due to their impact on living organisms, they are classified as endocrine disruptors acting on the hormonal system [3]. Despite restrictions on the use of BPA and phthalates in some countries, their impact on humans remains an important health issue [1,2].

Bisphenol A is used in the production of many everyday objects such as bottles, food containers, tin cans, thermal paper, toys, clothes, and electronical elements [1]. BPA is considered one of the most widely produced synthetic substances worldwide, and its production reaches over 10 million tons per year [1,4]. Unfortunately, BPA leaches out of the plastics and penetrates the natural environment. The presence of this substance has been noted in surface and tin water, soil, air and plants around the world [1,4,5]. The scale of the problem may be evidenced by the fact that the presence of BPA has even been found in the polar regions, i.e., an environment exposed to little anthropogenic pollution [6].

BPA also penetrates the digestive system, skin and lungs of humans and animals [1,7]. The alimentary route is the most important, and therefore, BPA in food containers (from which it may directly penetrate food) presents the greatest health risk [1,4].

Due to its similarity to estrogen, BPA links to estrogen receptors and adversely affects various internal organs and systems. It has been shown that BPA impairs the function of the reproductive, nervous and endocrine systems, negatively impacts the heart, gastrointestinal tract and liver, and disturbs immune cell function [1,4]. Moreover, correlations between exposure to BPA and neoplasms, diabetes, obesity and neurodegenerative diseases have also been reported [4,8].

Phthalates are a group of synthetic substances of which more than 25 compounds have a commercial use [2]. The production of phthalates reaches about eight million metric tons per year [9] and they are present in products such as cosmetics, toys, food packaging, paints and clothes [2]. Like BPA, phthalates are ubiquitous in the environment; they penetrate organisms through the digestive tract, inhalation and dermal absorption and impair the function of various internal organs [2]. It has been found that phthalates adversely affect the reproductive organs and the nervous, endocrine and immune systems, and may increase the risk of diabetes, neoplasms and obesity [2,10,11].

Despite previous studies on the influence of BPA and phthalates on human and animal health, many aspects of the impact of these substances on living organisms remain unclear. Further research on the health effects of BPA and phthalates are needed to better understand the mechanisms of their toxic impact. Therefore, original research studies, epidemiological observations and review articles concerning the impact of BPA and phthalates on human and animal organisms are welcomed for the Special Issue entitled “Health Effects of Bisphenol and Phthalate Exposure”; it is hoped that this will improve existing knowledge on these endocrine disruptors and reduce their negative influence on living organisms in the future.
